# Transnational Healthcare Practices Among Afghan, Syrian, and Ukrainian Refugee Older Adults in the Greater Toronto Area: A Study Protocol

**DOI:** 10.3390/healthcare13202644

**Published:** 2025-10-21

**Authors:** Sepali Guruge, Lu Wang, Kateryna Metersky, Areej Al-Hamad, Zhixi Zhuang, Cristina Catallo, Hasina Amanzai, Lixia Yang, Yasin M. Yasin, Vathsala Illesinghe

**Affiliations:** 1 Daphne Cockwell School of Nursing, Toronto Metropolitan University, Toronto, ON M5B 2K3, Canada; kateryna.metersky@torontomu.ca (K.M.); areej.hamad@torontomu.ca (A.A.-H.); ccatallo@torontomu.ca (C.C.); hamanzai@torontomu.ca (H.A.); 2 Department of Geography and Environmental Stuides, Toronto Metropolitan University, Toronto, ON M5B 2K3, Canada; luwang@torontomu.ca; 3 School of Urban and Regional Planning, Toronto Metropolitan University, Toronto, ON M5B 2K3, Canada; zczhuang@torontomu.ca; 4 Department of Psychology, Toronto Metropolitan University, Toronto, ON M5B 2K3, Canada; lixiay@torontomu.ca; 5 Faculty of Nursing, University of New Brunswick, Room 203, MacLaggan Hall, Saint John, NB E3B 5A3, Canada; yasin.yasin@unb.ca; 6 Policy Studies Program, Toronto Metropolitan University, Toronto, ON M5B 2K3, Canada; vathsalai@torontomu.ca

**Keywords:** Afghan, Canada, refugee older adults, refugee health, Syrian, transnational healthcare practices, Ukrainian

## Abstract

The global population of older adults is growing rapidly, and refugees are now a significant proportion of the older adult population in Canada. Transnational healthcare practices (THPs)—seeking health information or services from the country of origin—may be an essential strategy used by refugee older adults in Canada, but few studies have explored this phenomenon. This is a protocol for a study, which is aimed at developing a comprehensive understanding of the role THPs play in the lives of older adults from three refugee groups (Afghan, Syrian and Ukrainian) (re)settled in the Greater Toronto Area (GTA), Canada. It will be informed by Constructivist Grounded Theory and will consist of three phases. Phase 1 will involve semi-structured individual interviews with Afghan, Syrian, and Ukrainian refugee older adults living in the GTA (*n* = 75–90) to explore their perspectives and experiences with various types of THP. Phase 2 will also involve semi-structured interviews with 75–90 refugee older adults from the three communities to examine the role of THP in stress, coping, and resilience in the context of health promotion, illness diagnosis, and disease management as well as the individual and contextual factors driving the use of THPs. Phase 3 will involve six focus groups (*n* = 36–48) with refugee older adults from these communities to explore what information, care, supports, technology, and services are needed to manage their health and illnesses locally. This project will advance knowledge in the areas of (re)settlement and integration, aging, local healthcare access, and THPs among refugee older adults in Canada. The findings will inform more effective integration policies and the delivery of efficient and equitable health information, care, support, technology, and services that address the healthcare needs of refugee older adults, enabling them to age well and age in place.

## 1. Introduction

Aging and international migration are two intersecting global trends that are reshaping demographic profiles in many countries. Older adults, defined as individuals who are 60 years of age and older, are the fastest-growing age group in Canada [1]. Immigrant and refugee older adults now represent approximately 30% of the older adult population in Canada, and this proportion is even higher in urban centers such as Toronto, where about 70% of the older adult population is composed of immigrants and refugees [1]. Scholars are now focusing more on aging in the post-migration context, and research suggests that refugee older adults in particular tend to face inequities in accessing healthcare locally [2,3]. Having timely and easy access to the required health, social, and settlement services is an essential element of aging well and aging in place.

Transnational healthcare practices (THPs) are one response to unmet healthcare needs related to language differences, geographic access issues, high costs, lengthy wait times, and perceptions of poor service quality [4,5,6,7,8]. THPs can also be seen as an indicator of resourcefulness among older adults [9]. We define THPs as practices involving individuals living outside of their country of origin seeking healthcare from their country of origin through physical or other means [10]. THPs can be travel- or non-travel-based. The former involves a return to the home country to access healthcare [11] while non-travel based THPs include practices such as importing Western or Eastern or alternative medications and other medicinal products from a home country and/or virtually consulting healthcare providers in the country of origin [5,7,12].

Few studies have explored THPs among refugees in Canada and elsewhere, and even fewer have focused on these issues among refugee older adults. Refugees are forced to migrate, often due to war and other forms of civil, political, social, environmental, and economic unrest. They tend to be highly resilient, but often face considerable physical and mental health challenges [13] due to trauma and the short- and long-term conditions within which they must live. They also face major barriers to (re)settling in a new country, including language barriers, unfamiliar weather, un/under-employment and deskilling, and loss of social support [14], all of which can be exacerbated by experiences of discrimination and racism [15,16,17]. Refugees often face insurmountable barriers to obtaining health-related support, advice, and information, forcing many aging refugees to engage in THPs. This context underscores the significance of this study to examine the nature and extent of THPs among refugee older adults and explore how best to provide local access to timely and appropriate healthcare, information, and services.

## 2. Populations of Interest

Afghan, Syrian, and Ukrainian refugees are among the top five source countries of refugees currently arriving in Canada [18]. These groups have strong cross-border connections that can strongly influence the use of THPs.

Ukrainians face among Europe’s highest rates of chronic health conditions and have the lowest vaccination coverage [19]. The country also has Europe’s highest suicide rate at 30.6 deaths per 100,000 people, nearly three times the global average of 10.4 [20]. Many older Ukrainians struggle with serious health issues that limit mobility, and these problems are worsened during displacement, leading to reduced access to medications, medical treatment, and ongoing care and support [21]. Afghan older adults carry trauma from over four decades of political unrest and warfare, with displacement occurring through four major waves from the 1980s Soviet–Afghan war through the 2021 U.S. withdrawal [22]. After resettlement, both populations experience compounded difficulties. Many Ukrainian older adults struggle with mobility-limiting health issues that worsen during displacement, reducing access to medications and ongoing care [23]. Syrian refugee older adults in Canada face multiple forms of discrimination, insufficient income, inadequate shelter, and limited access to healthcare, food, and household necessities [24]. Few studies have explored mental health concerns among older Syrian refugees [23], although some reports suggest that 65% of older Syrian refugees exhibit signs of psychological distress [25]. These conditions, combined with harsh winters, contribute to both physical and mental health problems, which persist long after initial (re)settlement in host countries like Canada [25].

The THPs of Afghan, Syrian, and Ukrainian refugees are shaped by their varying socio-political contexts, which determine their migration pathways, reception, and access to housing and other services in Canada, as well as public opinions and discourses influenced by factors such as racialized status and religion.

This is evident in Canada’s response to different refugee crises; for example, the “unlimited number” of Ukrainians welcomed to Canada is compared to only 13,050 of the promised 40,000 Afghan refugees resettled, which is termed as the “hierarchy of deservingness” that privileges certain refugee populations over others based on various socio-political factors [26]. This is in direct contrast to the structure of the Canadian healthcare system that aims to serve all. However, we recognize the current healthcare system is strained, and it will be interesting to identify whether or not we see what structural barriers or hierarchies are developing as these groups navigate access to and receipt of healthcare. For example, Lipinski et al. (2022) highlight how differential treatment in host countries creates distinct pathways to healthcare access; Ukrainian refugees benefiting from ‘emergency travel authorizations’ as temporary migrants rather than refugees and receiving immediate processing and access to healthcare, while Afghan and Syrian refugees were accepted as refugees and navigate more restrictive bureaucratic processes that delay access to essential services [27].

Refugees’ experiences cannot be understood solely through healthcare access; it is also deeply shaped by host-society attitudes, political debates, and discrimination dynamics. Ozaydin’s (2018) analysis of anti-migrant sentiments in Turkey, a country hosting more than 3 million Syrian refugees, provides insights into how domestic political debates can fragment public opinion on refugee rights [28]. The negative public opinion created barriers to healthcare access, as it increased stigmatization of Syrian refugees seeking healthcare and reduced political support for comprehensive refugee healthcare programs.

Ökten Sipahioğlu’s (2023) comparative analysis of Syrian and Ukrainian refugee labeling provides a framework for understanding differential treatments of different groups [29]. Distinct labeling processes that categorize groups determine who deserves support/refuge. Comparing Ukrainian and Syrian refugees, these authors demonstrate how racialized statuses are being used to determine who receives more favorable treatment than others. Differences in the reception of refugees across European countries are discussed using media coverage and political discourse as examples. De Coninck (2022) makes similar comparisons between the differential treatment of Ukrainian and Afghan refugees in Europe [30].

It is important to capture the intersection of factors such as institutional discrimination, cultural barriers, and loss of support networks that creates conditions where refugees face worse health outcomes in destination countries [31], necessitating THPs that may include seeking medical consultations via digital platforms with homeland providers, importing medications from origin countries, or relying on diaspora networks for health information and support [31]. Understanding these socio-political dimensions is crucial for examining how refugees navigate healthcare systems post-migration and how THPs emerge as adaptive responses to structural inequities in healthcare access in their host countries.

## 3. Study Purpose and Research Questions

This paper presents a protocol for a study that will generate a comprehensive understanding of Afghan, Syrian, and Ukrainian refugee older adults’ engagement in and experiences of THPs in the Greater Toronto Area (GTA).

We will focus on the following research questions:

(1) What are Afghan, Syrian, and Ukrainian refugee older adults’ perspectives of and experiences with various types of THPs?

(2) How do THPs shape stress, coping, and resilience in the context of health promotion, illness diagnosis and prevention, and disease management among Afghan, Syrian, and Ukrainian refugee older adults?

(3) What individual and contextual factors and processes shape engagement in THPs among Afghan, Syrian, and Ukrainian refugee older adults?

(4) What health-related information, care, support, technology, and services do Afghan, Syrian, and Ukrainian refugee older adults require in order to promote their health and well-being and manage their healthcare needs locally?

## 4. Methods

This study will draw from constructivist grounded theory (CGT) [32], a qualitative inductive method that builds on the classical grounded theory developed by Glaser and Strauss in 1967, while incorporating interpretive and constructivist epistemologies. It is particularly well-suited for exploring complex social processes, worldviews, and multiple realities [32]. It combines interpretivism, critical theory, and social constructivism; researchers bring their own perspectives, experiences, and theoretical sensitivities to the research process, shaping how data are collected and analyzed [32]. CGT emphasizes the co-construction of knowledge between researchers and participants and allows theories to emerge from ongoing interactions between data and the researchers’ interpretive framework. It involves an iterative process of constant comparison, theoretical sampling, and memo-writing, enabling researchers to develop substantive theoretical understandings grounded in the experiences of participants while remaining reflexive about their own role in knowledge construction [32]. Researchers using CGT recognize the subjective nature of interpretation and the importance of context, making this approach particularly valuable for clarifying complex social phenomena and the lived experiences of groups [32]. In the context of this project, CGT will be particularly valuable as the research team examines how local and transnational contexts enable, hinder, or facilitate access to health information, support, services, and programs among older refugees—both within Canada and across transnational networks. The proposed project will also draw from social constructivism, which provides a lens to explore how contexts and cultural environments shape shared understandings of phenomena [33].

### 4.1. Study Design

We will use a cross-sectional study design with multiple phases to enable iterative and emergent design. Participants will be involved in two rounds of individual interviews followed by a focus group discussion. An advisory committee involving key community members from each targeted population group (Afghan; Syrian; and Ukrainian refugee older adults) will provide advice to ensure the research process is culturally appropriate.

### 4.2. Sample and Setting

A purposeful sample of Afghan, Syrian, and Ukrainian refugee older adults will be selected if they meet the following inclusion criteria: (1) 55 years of age or older; (2) born outside of Canada; (3) self-identifying as belonging to one of the three refugee communities; (4) living in the community (i.e., not in institutional settings) in the GTA; (5) have accessed any form of healthcare or related information or services from their home country since arriving in Canada; and (6) able to provide written or oral (informed) consent to participate in the study. We use 55 as the age criterion due to the short-term life expectancy in developing countries and different perceptions and expectations of aging and old age.

Multiple strategies will be used to address the language barrier. For example, bicultural and bilingual research assistants trained in interviewing/data collection, confidentiality, privacy, and data management will be recruited. They will also support translation of all study documents, verified by others who speak the language which includes research team members. Where feasible, the research assistants who conducted the interviews in will also transcribe and translate the interviews, ensuring that the cultural nuances are captured.

### 4.3. Sampling Method

We will use a combination of purposive and theoretical sampling [34]. Purposive sampling is suited for a CGT study as it allows researchers to select participants who have experienced the phenomenon of interest; thus, providing rich and relevant data. While it is recognized that these groups are substantially different in terms of their cultural, political, religious, and/or socioeconomic experiences, one of the propositions that we are trying to uncover is whether or not there are shared experiences related to THPs that transcend borders and ethnicities. We will also look at diversity and account for this in our sampling and analysis methods. To account for the intra-group variation within Afghan, Syrian, and Ukrainian refugee populations, this study will employ purposive sampling strategies to capture diversity across demographic characteristics and lived experiences within each group. During purposive sampling, participants will be recruited to ensure diversity based on gender, ethnicity, religious affiliation, socioeconomic background, educational attainment, length of time in the host country, and urban versus rural origins, while also considering factors such as family composition and pre-migration experiences.

Given the intra and inter group differences among the three target groups, we will include 25–30 members of each group in the sample. Theoretical saturation in grounded theory is not based on a numeric target but guided by the emergence of categories. Previous studies and papers suggest that a sample in the range of 20–40 can be sufficient for many grounded theory studies to reach saturation [32]. A sample of 30 is commonly recommended as a workable size that supports theoretical sampling and the development of robust categories without producing large amounts of data that is difficult to manage [35,36].

Using the inclusion criteria outlined earlier, we will recruit study participants who have engaged in THPs and can provide insights into specific aspects of the phenomenon under study [32]. Next, consistent with the iterative and emergent design of CGT, sampling criteria will be modified based on theoretical insights that develop during data collection and analysis [32]. For example, if initial interviews reveal that the length of resettlement period significantly influences THPs among refugee older adults, we will include participants with varying durations of residence to explore this dimension more thoroughly.

### 4.4. Participant Recruitment

After obtaining approvals from institutional research ethics boards, the research team will engage in a combination of active and passive strategies to recruit study participants. Examples of active recruitment strategies include asking community leaders or service providers to introduce the study at various community events or asking refugee older adults who participated in the study to refer others they know from their community. Examples of passive recruitment strategies may include posting flyers in relevant neighborhoods, libraries, and ethnic grocery stores. These strategies have proven to be successful in recruiting refugee and immigrant older adults in previous studies [37,38,39].

Potential participants will be asked to provide informed consent: they will receive information about the study and what participation entails, their rights, as well as the benefits and risks of participation. They will also receive information about honoraria and the right to withdraw from the study within two weeks of participation and have their information removed if they decide to stop participating.

Eligible participants will be asked to provide a separate consent to be re-contacted for participation in the subsequent phase(s) of the study. All contact information will be recorded separately from interview transcripts and will be used solely for the purpose of recruitment for the remaining phases of data collection. After participants have completed all phases for which they have provided consent, their contact information will be deleted from records within four weeks.

### 4.5. Data Collection

Phases 1 and 2 will include in-depth individual interviews, lasting approximately 45–60 min. Individual interviews will be conducted using a flexible interview guide [32] that allows modifications to the interview guide based on ideas emerging during completed interviews [32]. Based on the age of interviewees and the sensitive nature of information to be discussed, such as the challenges they face as refugees in Canada in accessing healthcare, we expect that some participants may become emotionally and/or physically tired after about one hour of interview time, so we will use multiple points of data collection to amass a large quantity of information. Phase 3 will involve six focus groups with (mostly) the same participants to confirm and fill any gaps in relation to the emerging theoretical understanding of the THPs. Each focus group will last approximately 60–90 min to ensure each participant’s voice is heard within the collective discussion. Focus groups can yield large amounts of data within a short time period and are particularly effective in reaching group consensus about gaps in previously collected data. They will also serve as a point for member checking to enhance the rigor of the study and validate the emergent results [32].

Phase 1: Individual interviews will be conducted by trained bilingual and bicultural research assistants. Interviews will be scheduled at a location and time and in the language of participants’ choice. As noted earlier, we expect to recruit 25–30 older refugees from each community for a total of 75–90 participants. Phase 1 interviews will clarify how Afghan, Syrian, and Ukrainian refugee older adults living in the GTA perceive THPs, their experiences, and the effectiveness of various types of THPs. The interview guide is attached as Supplementary Material (S1). The semi-structured interview guide will present the concepts of THP to the participants including the definition of THP and questions related to the practices that participants may or may not currently use. Because data collection and analysis occur simultaneously, we will modify the interview guide to address new ideas and patterns that emerge, concepts related to less visible issues like reliance on family networks or other nuances unique to this topic.

To ensure sample diversity and provide important contextual information during data analysis, we will collect some demographic information at the start of the interview, including age, ethnicity, gender, family composition, religious affiliation, education level, citizenship/immigration status, income, and length of stay in Canada. Participants who consent to participate in subsequent phases of the study will be asked to provide contact information.

Phase 2: About six months after the first set of interviews, the second set of individual interviews will be conducted with a total of 75–90 participants (25–30 from each community). We will include those who participated in Phase 1 and consented to be re-contacted. Additional participants will be recruited (if needed) using the same recruitment strategies as in Phase 1. These interviews will explore the role of THPs in stress perception, coping, and resilience in the context of health promotion, illness diagnosis, and disease management as well as what individual (e.g., age, gender, education) and contextual factors (e.g., local and transnational access to same-language physicians) and processes affect (force/promote/prevent) their access to and use of THPs. The interview guide is attached as Supplementary Material S2.

Phase 3: Six focus groups will be conducted with a subset of the participants who were interviewed in Phases 1 and 2. Two focus groups (each with 6–8 participants) will be held for each of the three communities for a total sample of about 36–48 participants. These focus groups will build on the results of the previous two phases: we will share the emerging findings with participants and ask them what health information, care, support, technology (including social media and internet use) and services they need in the GTA to manage their health and well-being locally, rather than engaging in THPs. A sample focus group discussion guide is included in Supplementary Material S3.

### 4.6. Data Analysis and Theory Development

As noted above, in each phase we will engage in concurrent participant recruitment, data collection, and data analysis. For this purpose, we will translate and transcribe each interview as soon as possible after the interview and subject the transcript to preliminary analysis before conducting the next set of interviews. This will allow time and space for any needed modifications to subsequent interview/discussion guides based on the collected data and preliminary analyses.

Initial stage of data analysis will involve line-by-line coding of interview transcripts; the most frequent initial codes will be developed into focused codes. Analyses will remain open to emerging codes while staying close to the words and experiences of participants. During the coding process, we will pay careful attention to the actions, meanings, and assumptions [32] related to refugee older adults’ engagement in THPs. We will use a constant comparative approach to data analysis, which involves continuously comparing data within and across cases, incidents, and processes, to compare how different refugee older adults navigate THPs; specifically, we will examine variations by gender, ethnicity, country of origin, health conditions, and other key demographic factors. The comparison of local and current practices with pre-migration healthcare experiences and THPs will help clarify continuities and adaptations. This iterative comparison will help us collate related codes into broader conceptual categories. We will recruit additional participants to fill gaps in and refine emerging theoretical ideas, using theoretical sampling until theoretical saturation is achieved. Analytical memos will be used to capture emerging insights, connections between ideas, and reflexive observations about the research process and findings throughout the coding process [40]. These memos will likely serve as crucial building blocks for theory development.

After identifying core categories that explain the main concerns and processes involved in THPs, we will develop a theoretical framework that explains how various factors (individual, familial, community, structural) interact to shape access to and engagement with THPs, and perhaps how they unfold over time and across different contexts, potentially culminating in a substantive theory about THPs among refugee older adults. This will involve drawing from the existing body of literature about refugee health and THPs to substantiate the emerging theory within a broader context to refine the theory.

We will compare how intra-group variations shape participants’ narratives, avoiding homogenizing experiences of diverse Afghan, Syrian, and Ukrainian refugee older adults. We will pay attention to intra-group variations during data coding and thematic development, and while identifying shared experiences, and the findings will be presented, with careful attention to the heterogeneity of experiences within each population group is included.

We will define saturation based on conceptual saturation when no new properties or dimensions of existing categories emerge. Practically, this involves examining thematic redundancy when new transcripts repeat themes already identified, no new codes or subcategories emerge, and code definitions and category structures stop evolving. We will predefine a stopping criterion, e.g., after 6–8 interviews yield no new themes, or when saturation is achieved across key theoretical constructs.

## 5. Discussion

This study will provide evidence to understand how various factors may support or inhibit THPs including the varied contexts, structural barriers, and individual, family, and/or community level networks and support.

Some studies have focused on how refugees maintain transnational connections [41,42], which can help clarify some aspects of THPs, but they have not provided direct evidence about refugee older adults, and their health behaviors, healthcare access, or health outcomes. The absence of refugee health-focused theoretical frameworks in the broader health systems research literature presents an opportunity for CGT work in this domain. While our specific protocol is focused on three refugee groups, the methods and results are likely to inform applications for others in similar contexts and settings.

Understanding the complex, culturally embedded, and contextual nature of health-seeking behaviors across borders lends itself to qualitative approaches, particularly in-depth interviews and focus groups. In-depth interviews have been particularly valuable in previous studies on THPs, capturing the nuanced ways migrants navigate between different healthcare systems, traditional healing practices, and cultural understandings of health and illness [43]. In-depth interviews and focus groups allow researchers to gather detailed and rich data about experiences, beliefs, and perspectives while exploring complex and sensitive topics [44,45].

The application of CGT to this topic offers several distinct methodological advantages. CGT helps explore phenomena from the perspectives of participants, making it well-suited for an exploration of how refugee older adults navigate (multiple) healthcare systems while capturing the intra and inter groups variations in experiences shaped by socio-political contexts and cultural beliefs and practices. Unlike predetermined theoretical frameworks that might impose biomedical assumptions based on norms in the host country, CGT can enable understandings of health, illness, and healing to emerge organically from the data [32]. This is particularly valuable when studying populations whose healthcare concepts and practices may differ significantly from those in the host country and among each other. Generating theory from empirical data, rather than applying existing theories, can help ensure that our results are directly relevant to the unique context of refugee healthcare experiences.

The constructivist epistemology of CGT explicitly acknowledges power relationships, which makes it appropriate for research involving vulnerable populations including refugee older adults. Its reflexive approach helps us to capture the researchers’ positions of power and privilege relative to research participants. This includes a critical examination of how structural inequalities including host-society attitudes and narratives about deservedness of different refugee groups shape healthcare access among refugee older adults. This attention to power dynamics is particularly valuable when engaging in research involving studying populations who may have experienced trauma and exclusion from services. The iterative design of CGT allows theoretical categories to emerge from data to capture the heterogeneous groups’ experiences based on country of origin, nature of conflict/displacement, migration trajectory, resettlement duration, legal status, and individual circumstances. The iterative design will also help clarify how healthcare behaviors evolve from pre-migration experiences through the resettlement and integration phases.

We expect to advance understanding of how structural factors intersect with individual agency in health decision-making, potentially influencing future research designs in migration and health studies.

## 6. Conclusions and Implications

Our findings about older Afghan, Syrian, and Ukrainian refugees may be relevant to other groups experiencing healthcare system disruption or change. They may be extended to other cultural groups who have strong traditional health beliefs and practices, groups that face challenges in navigating language and cultural barriers in healthcare settings, and individuals who rely on family and community networks for health support. All this research is situated at the intersection of aging-related health needs with migration-related vulnerabilities. Previous research suggests that THPs may be common among various ethnic communities regardless of their specific migration pathway [46,47,48]. We expect that our findings will be transferable to refugee older adults who have been forced out of other conflict-affected regions and may face comparable challenges in accessing timely and appropriate healthcare in host countries [49].

We expect to generate substantive theory that clarifies health-seeking behaviors among refugee older adults, and the role of THPs in refugee health integration and (re)settlement. This knowledge will contribute to the broader fields of migration health, health services research, (re)settlement and integration, aging, refugee older adults, and THPs in host countries including Canada. The findings will be of particular interest to researchers, practitioners, policymakers, and community members and will lead to the development of more effective and integration policies and delivery of efficient and equitable health information, care, and services. Specifically, they will inform social, settlement, and healthcare practices and policies in host countries and yield evidence-based recommendations for supporting refugee health during resettlement and integration processes.

As the global population of older adults continues to grow, students in health, social services, policy, and settlement-related fields, such as social work, psychology, nursing, public policy, and migration studies need to be better prepared to work with diverse older refugee populations. Our findings are likely to inform the development of specialized training for community health workers and newcomer and refugee service providers in host countries to better help refugee older adults navigate settlement services and access healthcare systems. They are also likely to contribute to continuing education programs for existing healthcare providers, helping them become more aware, understand, and respond appropriately to THPs.

Finally, the knowledge generated will benefit refugee older adults themselves, their families, and organizations serving immigrants and refugees. By clarifying THPs, we will be able to capture important elements of health promotion and illness diagnosis, management, and prevention, and use the findings to promote better services, programs, and policies, particularly community-based health promotion programs that build on and utilize existing transnational networks.

## Data Availability

Consent for public use of data will not be obtained from the study participants so data will not be openly shared.

## Supplementary Materials

The following supporting information can be downloaded at: https://www.mdpi.com/article/10.3390/healthcare13202644/s1. Reference [50] is cited in the supplementary materials.
