# Supplementary material for: Transnational Healthcare Practices Among Afghan, Syrian, and Ukrainian Refugee Older Adults in the Greater Toronto Area: A Study Protocol
Source: Healthcare (Basel). 2025 Oct 21;13(20):2644. doi: 10.3390/healthcare13202644 (PMC12562360; doi:10.3390/healthcare13202644)
Supplement: Supplementary file 1 [file healthcare-13-02644-s001.zip › healthcare-3825189-supplementary.pdf]

Supplementary File S1:

Transnational health care practices among Afghan, Syrian, and Ukrainian refugee older adults in the Greater Toronto Area – Individual Interview Guide for Phase 1

1. First, please tell me a bit about yourself. (Probes: age, gender, place of birth, places lived before arriving in Canada, length of stay in Canada, immigration status, employment/work)

2. Next, I would like to ask you about your own health and health management.
   - How would you rate your current health status? How has your overall physical and mental health status changed since coming to Canada? (Probes: If your health has declined/improved/remained stable, why? What are some of the things that you think may have contributed to these changes?)
   - What are some of the changes in your lifestyle (diet, outdoor activity and exercise routine, smoking, drinking)? How have these resulted in changes to your health or general well-being?
   - Do you visit doctors more/less frequently as compared to the time before you arrived in Canada? What is that?
   - What are some of the health problems you now have that require help from a doctor or nurse practitioner here in Canada?

3. Next, I would like to ask you about your experience in accessing healthcare services in the Greater Toronto area.
   - Where or how do you get help from a doctor or nurse practitioner, if you need it?
   - Describe your experience with your family doctor, nurse practitioner or other healthcare providers? (Probes: Where do you typically seek care? Which healthcare providers do you regularly access? How do you interact with these healthcare providers
   - How satisfied are you with your current family doctor/ nurse practitioner? Please give me an example of a good experience you have had with a doctor, nurse, or clinic here in Canada.
   - What inconveniences do you face in seeing your healthcare practitioner more regularly or as health concerns arise for you? (Probes: Language and/or cultural barriers, clinic/doctor location, travel distance and time, type of

transportation use, wait times at the clinic, costs of care/services, availability of health insurance and drug plans?)
  - What would make getting healthcare here easier for you?

4. What specific traditions from your culture do you think about when you seek help from a doctor here in Canada?

5. What kind of traditional/alternative healthcare providers do you see in the Greater Toronto Area (e.g. traditional healers, herbalist doctors, acupuncturists, chiropractors)
   - What do you use these healthcare providers for?
   - How would you describe your experience with them?
   - What is your level of satisfaction with these services?
   - How do they meet needs related to care and treatment?
   - What inconveniences do you face in seeing them more regularly or as health concerns arise for you? (Probes: Language and/or cultural barriers, clinic/doctor location, travel distance and time, type of transportation use, wait times at the clinic, costs of care/services, availability of health insurance and drug plans?)

6. Sometimes people rely on health-related advice and information from their home country while living in Canada. Tell us about your experiences in doing that.
   - What types of health-related information and advice have you accessed from your home country?
   - Who helps you to speak to or connect with a healthcare provider back home and how do they help?
   - What health-related websites that are unique to your home country do you access?

7. If you use healthcare services in Canada and from your home country, how do you decide which one to rely on for what health issues?

8. What experiences have you had with doctors or nurse practitioners in Canada who ask about and support your use of health care practices, resources, and services from your home country?

9. Besides the medicines that your doctor gives you in Canada, what other medicines, vitamins, herbs, teas, etc., do you take on your own and for what reasons?

10. What medicines, if any, have you or your family, friends or others brought for you from your home country to help manage your health? (Probe: antibiotics and traditional medicine like plants, herbs, teas, vitamins, supplements, etc.). How else do you get these medications (e.g., imported by others and purchased in Canada)?

11. What healthcare services, if any, have you sought when you went back to your home country (e.g., checkup, treatment, prescription, surgery, dental, vision care, herbalist doctors, acupuncturists, mental health-related)? Why did you decide to seek care in your home country. (Probes: Cost: insurance, drug plan, etc.; quality; availability in Canada; satisfaction with service; and perceived barriers?

12. What has been your experience seeking healthcare services or resources using online or virtual platforms (e.g., online doctor websites, video calls/FaceTime, telephone, texting, social media such as Facebook or Instagram, chat apps such as WhatsApp or WeChat)? Which of these are in your own language or from your country?

Probes:

- How did/do you find these resources or supports?
- Which of these are you currently using?
- Do you use any websites, social media platforms, or apps that are in your own language or from your home country?
- Do you follow certain people who give health advice, or are you in any online health groups?
- What are the main reasons for seeking these services?
- How satisfied are you with such services?
- What helps you access these resources, and what prevents you from using them?

13. Now I would like to ask you about your social connections with those in your home country.
    - How do you keep in touch with family, friends, and others from your home country?
    - How frequently do you communicate with them? How do you talk to them?
    - What social media (e.g., Facebook, Instagram) or chat apps (e.g., WhatsApp, WeChat), if any, do you use to stay in contact? How do you use these tools (e.g. to share pictures, videos, talk)?
    - How often do you travel to your home country? What are some reasons for travelling to your country? How long do you usually stay there?

14. Now I would like to ask you about the federal, provincial, and community supports in the Toronto area.
    - To your knowledge, are there any government supports for Afghan/Syrian/Ukrainian refugees seeking healthcare services in Toronto (e.g., social workers, information centers, or monetary stipends)? If yes, what are they?
    - Do you know how to look for this information? If yes, what are some of the sources? Are these useful/helpful? Please elaborate on your response.
    - How do those supports reduce your need to seek health care from your home country?

[Brief Resilience Scale (BRS)[1]]

15. Lastly, I would like to ask you a few questions about your response to difficulties faced in everyday life. On a scale of 1 to 5, with 1 being Strongly Disagree and 5 being Strongly Agree, would you say:
    - You tend to bounce back quickly after hard times.
    - You have a hard time making it through stressful events.
    - It does not take you long to recover from a stressful event.
    - It is hard for you to snap back when something bad happens.
    - You usually come through difficult times with little trouble.
    - You tend to take a long time to get over setbacks in your life.

Is there anything else that you would like to add to what we talked about?

[1] Smith, B. W., Dalen, J., Wiggins, K., Tooley, E., Christopher, P., & Bernard, J. (2008). The Brief Resilience Scale: Assessing the ability to bounce back. International Journal of Behavioral Medicine, 15(3), 194–200.

Supplementary File S2:

Transnational health care practices among Afghan, Syrian, and Ukrainian refugee older adults in the Greater Toronto Area: Individual Interview - Guide for Phase 2

[First-time participants only]

1. First, can you tell me a bit about yourself? (Probes: Age, gender, place of birth and places lived before arriving in Canada, length of stay in Canada, immigration status, employment/work.)

Next, I would like to ask you about your general health and health management.

- How would you rate your current health status? How has your overall physical and mental health status changed since coming to Canada? (Probe: If health has declined/improved/remained stable, what do you think may have contributed to this change?)
- Do you visit doctors more/less frequently as compared to the time before you arrived in Canada? What is the reason for this change?
- What are some of the changes in your lifestyle (diet, outdoor activity and exercise routine, smoking, drinking)? How have these resulted in changes to your health or general well-being?
- What are some of the health problems you now have that require help from a doctor or nurse practitioner here in Canada?

(For the individuals who participated in Phase 1]

(record any changes to their demographic information such as those related to immigration status, employment/work, and avoid repetitious questions)

1. In your own opinion, how is your overall health? What has changed since the last time we met?
   Have you used healthcare from your home country more or less often since we last met? What type? Why?

Next, we will talk about accessing healthcare in Canada.

2. What health screening and/or preventative care do you do to stay healthy?

- Tell me about a time you participated in health screenings or preventive care in Canada (for example screening for heart disease, colon/breast /prostate cancer?
- Tell me about a time when you participated in such health screenings or preventative care in your home country?
- How do you decide which preventive health measures to follow when you receive different recommendations?
- What foods, medicines, or exercises do you use top promote health?
- How often do you tell your doctors or nurses here about treatments from home country? What information do you share normally? What are their reactions?

3. What are your biggest worries about getting health care as an older adult living in a new country?
    - Please explain in detail when and how this worry affects you? What would help you worry less?
    - How are these things different back home?
    - Please tell me about a time when you had to choose between getting care in Canada and getting care from your home country?

4. How do you feel when you need to deal with doctors and hospitals in Canada vs. your home country?
    - Where (which country) do you feel more comfortable? What makes you feel nervous or safe?
    - Tell me about a time when you felt really good or really bad at a doctor's or hospital visit.
    - How do you feel when the doctor doesn't speak your language? How do these feelings affect your health or make you avoid doctors?

Healthcare Decision-Making and Information Sources

5. When you have health problems, where do you typically seek care first: Canada, your country of origin, or do you consult multiple sources at the same time? Please explain your decision-making process.

6. How do you get information about health care? (Probe: Do you ask doctors here, family/resources back home, or look online?)

- Which sources do you trust the most? Why? Please give me an example of how and when you used it?

- Who in your family or community gives you health advice? What type of advice do you get or seek from them?

7. How do you decide whether to trust advice from healthcare providers in Canada or those in your country of origin?
    - Have you ever refused treatment or advice from a healthcare provider in Canada and why?

   What role do family members, friends, and others in your home country play in helping you make health decisions? Can you give me an example? Who do you think has the most impact on your decisions and why?

8. What other sources of information do you seek (Probe: look online, specific websites etc.)?

   Things that may affect healthcare decisions

9. What do you think the care you received depends on? (Probe: based on age, gender, education, language skills or something else)
    - Do you feel healthcare providers treat you differently as a result? How?
    - Which of these things make it harder or easier to get health care here in Canada compared to back home? How?

10. What makes it hard for you to get healthcare in Canada and in your home country? (Probes: Money, language, legal status, travel, paperwork, or something else?)
    - Tell me about a time when you could not get health care or medicine/surgery or something else like dental or vision care because you did not have money to pay for it. What did you do about it?
    - Tell me about a time that you did not get care because you could not travel to your home country. What did you do about it? How did you access care?
    - Was there any time that paperwork or legal problems prevented you from getting care/treatment/medicine? What happened in that situation?
    - How does insurance affect access here in Canada and in your home country?
    - Have you ever traveled to home country for health care? What makes it easy/difficult to get health care including medicines and health procedures in person in your home country?

Coping, Resilience, and Support Systems

11. What coping strategies or resources do you wish you had better access to - in Canada and in your home country?
    - What are some coping strategies/resources that you have here that you did not have back home? What are the resources you had back home that you don't have now?
    - How do you manage your fears and concerns about accessing health care here and back home?
    - How have you helped others (family, friends, others) cope with their fears and concerns?
    - How does your family network (both here and back home) support you?

12. What gives you the most strength and support when dealing with serious health problems and why?
    - How important is it for you to maintain health practices from your home country? Why?

Discrimination and Cultural Factors

13. Have you ever felt disrespected or treated unfairly here because of your background? Please give me an example or two.
    - What did you do in those situations?
    - What changes have you made to how or where you access health care, because of these experiences?
    - What similar experiences have you had in your home country?
    - If not, what was different about those settings?
    - In your view, what could have been done differently to prevent you from being disrespected or treated unfairly?

14. Overall, how does your ability to access health care in both countries shape or affect your health outcomes?

15. What advice would you give to other older immigrants or refugees about managing health care across countries?

Is there anything else that you would like to add to what we talked about?

Supplementary File S3:

Transnational health care practices among Afghan, Syrian, and Ukrainian refugee older adults in the Greater Toronto Area – Focus Group Discussion Guide* for Phase 3

---

1. Describe what "being healthy" means to you and others in your community. How might this be different from the way Canadian healthcare providers see "being healthy"? (Probe: Think about physical health, mental wellbeing, spiritual health, etc.)

- Purpose: To understand culturally constructed meanings of health and wellness, and how these may differ from dominant medical models

2. Let us talk about a time when you felt truly cared for by a healthcare provider here in the Greater Toronto Area. What made that experience meaningful to you? Share as much detail as you're comfortable with - we want to understand what "good care" looks like through your eyes.

- Purpose: To explore participants' constructed meanings of quality care and significant healthcare experiences

3. Think about times when you have felt misunderstood or not heard by healthcare providers here. What do you think they were missing about you or your needs? (Probe: This could be about communication, cultural differences, assumptions, or anything that made you feel disconnected.)

- Purpose: To explore experiences where different constructions of reality collide, and understanding barriers to meaningful healthcare relationships

4. Thinking about your time since arriving in Canada, how has your understanding of the healthcare system here changed over time? What have you learned that you did not know before? (Probe: Consider both positive and negative experiences and challenging situations.)

- Purpose: To understand how meaning-making evolves through experience and interaction with healthcare systems

5. Tell me about the conversations you have had with family, friends, or community members about healthcare here. What stories do people share? What advice gets passed

around? (Probe: Think about both formal and informal discussions - at home, community centers, religious places, and social gatherings.)

- Purpose: To explore how healthcare knowledge and meanings are socially constructed and shared within communities

6. How would you describe to a new immigrant older adult from your country what healthcare is like here in the Greater Toronto Area? What story would you tell them? What would you want them to know? (Probe: Think about what you wish someone had told you when you first arrived.)

- Purpose: To understand how participants construct and communicate their healthcare reality to others, revealing core meanings and experiences

7. If you were talking to healthcare providers about caring for people from your community, what would you tell them? What would help them understand your community members' experiences and needs better? (Probe: Think about what they should know about your culture, experiences, values, or ways of understanding health.)

- Purpose: To allow participants to construct and articulate their expertise, and identify key cultural knowledge for healthcare provision

8. Describe your ideal healthcare experience here in the Greater Toronto Area. What would it look, feel, and sound like? (Probe: Think about all the details - the environment, how people interact with you, what happens, how you feel.)

- Purpose: To co-construct visions of ideal healthcare, allowing participants to articulate their values and needs through detailed description

9. Based on what we talked about here today, what feels most important to share with healthcare decision-makers?

- Purpose: To encourage reflection on the meaning-making process that occurred during the discussion and identifying key constructed understandings.

10. Let us take a moment to think about what resonated most with you today. What felt most important and relevant?

---

*The focus group discussion guide will be refined based on the results from Phases 1 and 2.
